# Long-term efficacy and reduced side-effects of buprenorphine in patients with moderate and severe chronic pain

**DOI:** 10.3389/fphar.2024.1454601

**Published:** 2024-08-08

**Authors:** Alfonso Papa, Anna Maria Salzano, Maria Teresa Di Dato, Vincenzo Desiderio, Pietro Buonavolontà, Pietro Mango, Elisabetta Saracco, Dario Tammaro, Livio Luongo, Sabatino Maione

**Affiliations:** ^1^ Department of Pain Management—AO “Ospedale dei Colli”–Monaldi Hospital, Napoli, Italy; ^2^ Department of Experimental Medicine, University of Campania “Luigi Vanvitelli”, Naples, Italy; ^3^ Department of Experimental Medicine, Division of Pharmacology, University of Campania “Luigi Vanvitelli”, Naples, Italy

**Keywords:** chronic pain, opioids, tolerance, transdermal patches, pain relief, opioid crisis

## Abstract

**Background:**

Chronic pain significantly impacts quality of life and poses substantial public health challenges. Buprenorphine, a synthetic analog of thebaine, is recognized for its potential in managing moderate to severe chronic pain with fewer side effects and a lower incidence of tolerance compared to traditional opioids.

**Objective:**

This retrospective study aimed to assess the long-term efficacy and safety of buprenorphine transdermal patches in patients with moderate and severe chronic pain, with a focus on pain relief sustainability and tolerance development.

**Methods:**

This retrospective observational study involved 246 patients prescribed buprenorphine transdermal patches. We evaluated changes in pain intensity using the Numeric Rating Scale (NRS), assessed opioid tolerance based on FDA guidelines for morphine-equivalent doses, and measured patient-reported outcomes through the Patients’ Global Impression of Change (PGIC). Any adverse events were also recorded.

**Results:**

Over the 36-month period, there was a significant reduction in NRS scores for both moderate and severe pain patients, demonstrating buprenorphine’s sustained analgesic effect. Tolerance measurement indicated that no patients required increases in morphine-equivalent doses that would meet or exceed the FDA’s threshold for opioid tolerance (60 mg/day of morphine or equivalent). Additionally, patient satisfaction was high, with the PGIC reflecting significant improvements in pain management and overall wellbeing. The side effects were minimal, with skin reactions and nausea being the most commonly reported but manageable adverse events.

**Conclusion:**

The study findings validate the long-term use of buprenorphine transdermal patches as an effective and safe option for chronic pain management, maintaining efficacy without significant tolerance development. These results support the continued and expanded use of buprenorphine in clinical settings, emphasizing its role in reducing the burdens of chronic pain and opioid-related side effects. Further research is encouraged to refine pain management protocols and explore buprenorphine’s full potential in diverse patient populations.

## 1 Introduction

Chronic pain represents a significant challenge in clinical management, often leading to compromised quality of life for patients. Recent estimates from the World Health Organization (WHO) indicate that chronic pain impacts approximately 20% of the world’s population, significantly impairing daily functionality, interpersonal relationships, and emotional wellbeing (Mills et al., 2019). In the United States, the economic burden of chronic pain, encompassing medical expenses and productivity losses, ranges between US$560 and US$635 billion annually (Nahin et al., 2023). Furthermore, chronic pain is associated with various comorbidities, including depression, anxiety, physical disability, hypo cognition and sleep disturbances, further contributing to the overall burden of the disease (Cohen et al., 2021).

Opioids still represent a cornerstone in the pharmacological armamentarium for treating moderate and severe chronic pain (Alorfi, 2023). Nevertheless, chronic opioid therapy with potent (schedule II) opioids like morphine, oxycodone, fentanyl, hydrocodone, and hydromorphone poses numerous challenges, including severe side effects such as hypogonadism, infections, immunosuppression, respiratory depression, and mortality (Holtman and Jellish, 2012; Poon et al., 2021). Additionally, gastrointestinal issues such as opioid-induced constipation (are highly prevalent, often emerging as a primary reason for discontinuing opioid therapy (Sizar et al., 2023).

Tolerance, along with the potential for abuse and dependence, represents another significant challenge in opioid therapy (Mercadante et al., 2019). Tolerance occurs when the efficacy of a drug diminishes over time, leading to the need for higher doses to achieve the same therapeutic effect. Mechanisms underlying opioid tolerance involve drug-induced adaptations or allostatic changes at various levels, including cellular, circuitry, and systemic levels (Aston-Jones and Harris, 2004; Christie, 2008). The development of tolerance presents a significant issue, as it demands higher opioid doses to achieve the same therapeutic effect. This phenomenon not only reduces the effectiveness of opioids but also raises the likelihood of experiencing withdrawal symptoms and developing addiction. Additionally, long-term opioid therapy may lead to dose escalation, potentially inducing opioid-induced hyperalgesia, characterized by increased sensitivity to painful stimuli, exacerbating rather than alleviating pain perception (Mercadante et al., 2019) The addictive nature of opioids has contributed to the opioid crisis, posing a substantial challenge to various sectors including social, economic, and public health not only in the United States but also in other countries (Jalali et al., 2020). Millions of prescription opioids are misused annually, resulting in significant financial costs and high overdose death rates. To address this crisis, the US Department of Health and Human Services established a task force promoting better pain management practices (Gudin and Fudin, 2020).

One recommended approach is the preferential use of buprenorphine, not only for opioid use disorder but also for pain management, suggesting it as a primary option when clinically indicated rather than a secondary choice after other opioids failure (Pergolizzi et al., 2008; Breivik, 2013; Ehrlich and Darcq, 2018).

Buprenorphine, a lipophilic opioid analgesic derived from thebaine, has emerged as a promising option due to its unique pharmacodynamics (Webster et al., 2020). Buprenorphine binds to mu (MOR), kappa (KOR), and delta (DOR) opioid receptors, exhibiting a sort of biased agonism towards MOR and antagonism towards KOR and DOR (Pergolizzi et al., 2010; Webster et al., 2020; Infantino et al., 2021). Additionally, it binds to opioid-like receptor 1 (OLR-1), the receptor for orphanin FQ/nociception (Pergolizzi et al., 2010; Infantino et al., 2021). The biased agonism of buprenorphine at MOR, coupled with its distinct pharmacokinetic properties, contributes to its efficacy and safety profile.

Buprenorphine exhibits unique binding characteristics, primarily binding to acidic glycoproteins like α1-acid glycoprotein (AGP) rather than extensively to plasma proteins such as albumin (Pergolizzi et al., 2010). This differential binding minimizes drug-drug interactions in the distribution phase and enhances bioavailability, making buprenorphine a preferred option in elderly patients, in which the levels of albumin are decreased without changes in the AGP (Pergolizzi et al., 2010; Infantino et al., 2021). The pharmacological advantages of buprenorphine extend to its metabolism and excretion, which are favorable in clinical practice. Being metabolized primarily by cytochrome (CYP) 3A4 to its metabolite norbuprenorphine, buprenorphine is associated with fewer drug interactions, in the metabolism phase, compared to other opioids (Zhang et al., 2003). This is due to the possibility to directly conjugate buprenorphine by skipping the CYP activity. Importantly, its minimal renal excretion renders it suitable for use in patients with renal failure without necessitating dosage adjustments (Owsiany et al., 2019; Zhuo et al., 2021).

Finally, in response to the opioid epidemic and the need for enhanced chronic pain management, new methods of drug administration have been developed, including tamper-resistant formulations. Among these innovations, the transdermal drug delivery system stands out as a notable advancement, offering distinct benefits compared to conventional routes like parenteral and oral administration (Alkilaniet al., 2015). Transdermal delivery avoids discomfort associated with injections and multiple oral doses, provides constant plasma drug concentrations, bypasses hepatic metabolism and poor absorption from the gastrointestinal tract, and enhances patient compliance by reducing the frequency of administration. Additionally, the risk of local adverse events is minimized as the site of drug delivery can be regularly changed (Evans and Easthope, 2003).

Considering these characteristics, buprenorphine transdermal patches emerge as a preferred option for managing chronic pain, particularly in populations such as the elderly and those with renal and hepatic impairments (Plosker, 2011). However, despite its potential benefits, there remains a need for further research to elucidate its full mechanisms of action and optimize its clinical use.

According to NIH guidelines, buprenorphine is mainly used to treat moderate to severe pain, as it exhibits only partial analgesic activity at the mu-opioid receptor and exhibits a ceiling effect (Kumar et al., 2023). However, buprenorphine shows a ceiling effect on those MORs involved in respiratory depression but not on the MORs active at the pain axis that drive the analgesia. Intriguingly, unlike traditional opioids, buprenorphine demonstrates biased agonism towards MOR, preferentially activating G protein-mediated signaling over β-arrestin-mediated signaling (Neto et al., 2020; Infantino et al., 2021). This selective activation results in analgesia with reduced side effects such as respiratory depression and constipation (Neto et al., 2020; Webster et al., 2020; Infantino et al., 2021). Hence, buprenorphine could be considered a frontline treatment option for individuals suffering from severe pain, particularly if it exhibits lower tolerance induction compared to alternative opioids.

By analyzing buprenorphine’s efficacy, tolerance induction, and side effects over a 3-year follow-up period in patients with moderate and severe chronic pain, this retrospective study aims to offer insights into the effectiveness of buprenorphine in managing long-term pain. These findings will provide valuable information to clinical practice, facilitating the optimization of patient care in this challenging population.

## 2 Materials and methods

### 2.1 Study design

This study is an investigator-initiated, monocentric, retrospective, observational study designed to assess the long-term efficacy and safety of buprenorphine in the management of moderate and severe chronic pain. The retrospective analysis focused on patients who were administered buprenorphine’s transdermal patches as part of their treatment regimen between January 2021 and January 2024 at the Pain Department of the Azienda Ospedaliera Specialistica dei Colli–Ospedale Monaldi Napoli, Italy.

The study received ethical approval from the Campania SUD Ethics Committee, approval number AOC/0011976/2024. Prior to participation, all patients provided informed consent. This study strictly adhered to the ethical standards of the committee responsible for human experimentation (institutional and national) and the Helsinki Declaration of 1975, as updated in 2008, ensuring the highest ethical considerations and patient safety throughout the study period.

### 2.2 Data source

Data were gathered from the electronic health records of patients who were prescribed buprenorphine as part of their chronic pain management strategy. The treatment regimen consisted of administering buprenorphine’s transdermal patches according to individual patient needs, as determined by their managing healthcare professionals at the Azienda Ospedaliera Specialistica dei Colli–Ospedale Monaldi, Napoli, Italy. The specific dosage and frequency of buprenorphine administration were tailored to the severity of the pain and the patient’s overall response to the treatment, adhering to standard clinical practices for pain management.

In addition to the investigational treatment data, all concurrent treatments provided alongside buprenorphine, as well as any treatments received within 7 days prior to the initiation of buprenorphine therapy, were documented. This included the type of medication, dosage, and administration dates and times. Demographic details, medical history, weight measurements, adverse events, and clinical outcomes were also systematically recorded.

Patients were followed for a period of 3 years. Comprehensive physical examinations and complete pain evaluations were conducted at the start of buprenorphine treatment and at 6, 12, 24 and 36 months, including assessments of pain symptoms, physical function, and overall wellbeing.

### 2.3 Inclusion and exclusion criteria

#### 2.3.1 Inclusion criteria

- Patients aged 18 years and above.
- Patients naïve to opioid.
- Diagnosis of moderate or severe chronic pain, originating from various etiologies, including but not limited to musculoskeletal, myofascial, rheumatic conditions, and neuropathic pain; the pain should have persisted for more than 3 months, aligning with the chronic pain definition.
- A baseline Numeric Rating Scale (NRS) score for pain greater than 5, indicating moderate to severe pain intensity.
- Treatment with buprenorphine for a minimum duration of 6 months.

#### 2.3.2 Exclusion criteria

- Oncological patients
- Patients receiving concurrent treatments that could influence the assessment of buprenorphine’s efficacy, such as other major opioids, or invasive therapies.
- Pregnant or breastfeeding women.
- Patients with a history of past opioid abuse.
- A known allergy or hypersensitivity to buprenorphine or any of its components.

### 2.4 Clinical investigation endpoints

The study meticulously tracked and analyzed patient outcomes and complications from medical records to assess the efficacy and safety of buprenorphine in chronic pain management.

#### 2.4.1 Primary efficacy endpoint

The reduction of pain in patients with moderate and severe chronic pain as quantified by changes in the Numeric Rating Scale (NRS) scores at each follow-up period compared to baseline, with a specific focus on the sustained efficacy over 36 months.

#### 2.4.2 Secondary efficacy endpoints

- The percentage of responders at each follow-up interval (6, 12, 24, and 36 months), where responders are defined by significant reductions in NRS scores (as defined in the statistical analysis section) and positive evaluations on the Patients’ Global Impression of Change (PGIC).
- Patient satisfaction and quality of life improvements, measured by PGIC and supplemented by detailed patient interviews and quality of life assessments to provide a comprehensive view of the treatment impact over time.
- Evaluation of opioid tolerance development, specifically assessing if patients required increasing doses of buprenorphine to achieve the same level of pain relief initially provided by the treatment over the 36-month period. Tolerance, as defined by the US Food and Drug Administration (FDA), is quantified by the rate of dosage increase required to maintain effective pain management, with a significant increase indicating the development of tolerance.

#### 2.4.3 Safety endpoint

Safety assessments focused on the documentation and analysis of adverse events (AEs). These events were coded using the Medical Dictionary for Regulatory Activities (MedDRA) version 16.0 and categorized by system organ class and preferred terminology (Harrison and Mozzicato, 2009). The analysis included a detailed summary of adverse drug effects (ADEs) directly attributable to buprenorphine, serious adverse events (SAEs), and any adverse events prompting discontinuation of the therapy, thereby offering a thorough evaluation of buprenorphine’s safety profile in the treatment regimen for moderate to severe chronic pain.

### 2.5 Buprenorphine’s transdermal patches

The study utilized buprenorphine transdermal patches, marketed under the commercial name Busette (Sandoz, Basel, Switzerland), for the management of moderate to severe chronic pain in adults. These patches are specifically designed for long-term pain that requires the use of a strong painkiller. Busette transdermal patches contains buprenorphine as the active ingredient, and the patches are designed for transdermal use. The patches have been applied to clean, intact, non-irritated skin on the upper torso, upper arm, or the upper back. Patients have been advised to change the patch every 7 days, preferably at the same time to maintain consistent pain relief. These patches have been chosen because they provide a controlled, steady release of buprenorphine, offering a manageable option for adults dealing with chronic pain.

### 2.6 Measurement of pain

The Numeric Rating Scale (NRS) is a widely utilized tool for pain assessment, renowned for its simplicity in administration. It is a unidimensional scale, designed exclusively to measure pain intensity without considering other factors. The NRS is a numeric scale ranging from 0 to 10, where each number represents a level of pain experienced by the patient: a score of 0 indicates no pain; a score of 10 represents the maximum imaginable pain; scores between 1 and 9 signify increasing levels of pain intensity. According to literature data, this study classifies patients with moderate pain as those with a NRS score ranging from 5 to 6. Patients experiencing severe pain are classified as those with an NRS score equal to or greater than 6 (McCormack et al., 1988; Hawker et al., 2011; Alschuler et al., 2012).

### 2.7 Measurement of patients’ degree of satisfaction

The Patients’ Global Impression of Change (PGIC) scale is specifically designed to assess patients’ perceptions of change following treatment, that is, whether they feel “better” or “worse.” (Eremenco et al., 2022) It is a 7-point verbal scale offering options ranging from “very much improved” to “very much worsened,” including “much improved,” “minimally improved,” “no change,” “minimally worsened,” and “much worsened.” The PGIC is widely used in clinical studies that assess pain relief following treatment due to its ease of administration, scoring simplicity, and because it is a generic scale applicable across a wide range of conditions and treatments.

### 2.8 Measurement of buprenorphine tolerance

The measurement of buprenorphine tolerance, administered via transdermal patches (Busette), was a crucial aspect of assessing long-term treatment efficacy for patients with moderate and severe chronic pain. The FDA defines opioid tolerance as the need for increasing doses of an opioid to maintain the same level of analgesia that could previously be achieved at lower doses, without the progression of the underlying cause of pain.According to the FDA, opioid tolerance is considered to have developed when a patient requires at least 60 mg/day of morphine or an equianalgesic dose of another opioid for at least 1 week (Dowell et al., 2022; Rx only, 2024). In contrast, analgesic tolerance refers to the general reduction in the effectiveness of any analgesic medication over time, necessitating higher doses to achieve the same level of pain relief. In this study, we focused on opioid tolerance, as defined by FDA (Dowell et al., 2022). To determine the development of tolerance to buprenorphine, we translated the dosages of buprenorphine administered to patients into morphine-equivalent doses using an equianalgesic conversion factor. According to the conversion factor provided by the FDA and other governmental organizations, 5 μg per hour of transdermal buprenorphine is equivalent to 12 mg per 24 h of morphine, and, thus, the threshold dose of 60 mg/day of morphine correspond to 25mcg/h of transdermal buprnorphine (Cancer et al., 2012, Care Network; Opioid conversion ratios Guidance document 2, 2021).

Therefore, we used the following formula to calculate the morphine-equivalent doses from the administered buprenorphine doses:

$$\text{Morphine Equivalent Dose }\left(\text{mg}\right)=\text{Buprenorphine Dosage }\left(\text{mcg}\right)\times \left(12/5\right)$$

For each patient, buprenorphine dosages at baseline, 6, 12, 24, and 36 months were recorded. These dosages were then converted to their morphine-equivalent using the above formula to assess if they reached or exceeded the 60 mg/day morphine threshold (or the 25mcg/h of transdermal buprenorphine threshold) indicative of opioid tolerance. The development of tolerance was evaluated by analyzing the trends in morphine-equivalent doses over time. An increasing trend in these doses approaching or surpassing the tolerance threshold would indicate the development of tolerance. Statistical methods used included linear regression to analyze the rate of increase in morphine-equivalent doses over time, with a focus on the slope of the regression line. A steeper slope would suggest a higher rate of tolerance development.

### 2.9 Statistical analysis

All analyses were conducted on an intent-to-treat basis, with missing data addressed using multiple imputation techniques to maintain the robustness of our findings. The promoter center’s database includes approximately 280 potentially selectable patients. Given the retrospective nature of the study, it was deemed appropriate to analyze the data from as many patients as possible.

The treatment effect is represented as follows: Prog = NRS outcome at 36 months of treatment; BAS = NRS outcome at baseline. The null hypothesis is represented by Prog = BAS, and the alternative hypothesis by Prog ≠ BAS. Statistical tests (paired samples Student’s t-test, two-tailed) have been conducted at a significance level of 0.05 to demonstrate improvement in scores after the treatment period. The paired Samples *t*-test (two tailed) was employed to compare the mean NRS scores at baseline and subsequent follow-up intervals (6, 12, 24, and 36 months). Prior to applying the paired samples *t*-test, data normality was assessed using the Shapiro-Wilk test, and homogeneity of variances was checked via Levene’s test. Where assumptions were not met, non-parametric alternatives were employed. The Sídák’s Multiple Comparisons Test was specifically used to adjust for multiple comparisons in scenarios where pain scores were compared across more than two time points. This approach helped to maintain the overall Type I error rate, providing a more stringent criterion for statistical significance, especially relevant in the assessments from baseline to subsequent follow-ups. The Chi-square Test for contingency tables was applied to evaluate the distribution of categorical data derived from the PGIC. The Mann-Whitney *U* Test was used to compare the distributions of PGIC scores between patients with moderate and severe pain. A linear regression analysis was employed to assess the rate of increase in morphine-equivalent doses over time, which is indicative of potential tolerance development. The slope of the regression line provided a quantifiable measure of how dosage requirements changed over the study period, offering insights into whether patients were developing tolerance to buprenorphine.

## 3 Results

### 3.1 Patients demographic

The study analyzed a cohort of 246 patients, consisting of 74 males and 172 females. The mean age was 70.13 years (SD = 8.45) for individuals experiencing moderate pain and 69.45 years (SD = 9.72) for those with severe pain, with ages ranging from a minimum of 55 to a maximum of 85 years across the participant group (Table 1). For the sake of clarity and to minimize potential confounding variables, only patients diagnosed with arthritis and arthritis-related pain were included in the study.

**TABLE 1:** Patient’s demographic data. The table shows the distribution of patients by severity of pain and gender, along with average age.

	Total number of patients	Male	Female	Mean age
Moderate pain	75	14	61	70.8
Severe pain	171	60	111	69.5

**FIGURE 1:**
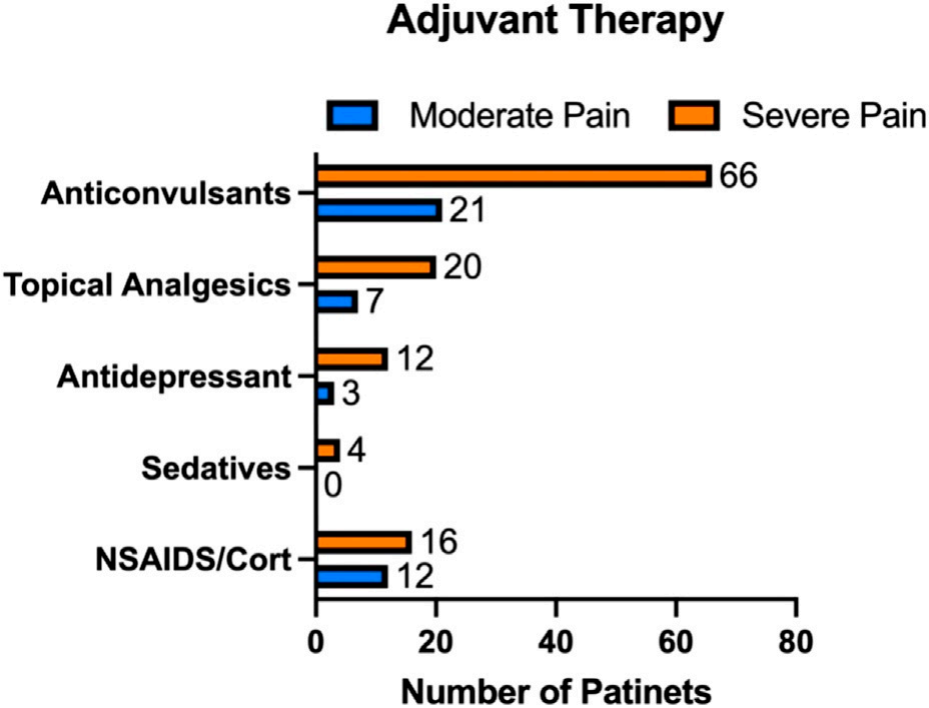
Additional therapies prescribed alongside buprenorphine for chronic pain management. Among the 248 patients enrolled, 118 with severe pain and 34 with moderate pain were prescribed adjuvant treatments, including anticonvulsants, topical agents, antidepressants, sedatives, and NSAIDs/corticosteroids.

In addition to buprenorphine, patients were often prescribed a variety of concomitant treatments to address their chronic pain and related conditions. Out of the 248 enrolled patients, 118 starting with severe pain and 34 with moderate pain were prescribed with adjuvant therapies. These included anticonvulsants, topical agents for localized pain relief, antidepressants, sedatives, and NSAIDs/corticosteroids (Figure 1).

### 3.2 NRS score in patients with moderate pain

**FIGURE 2:**
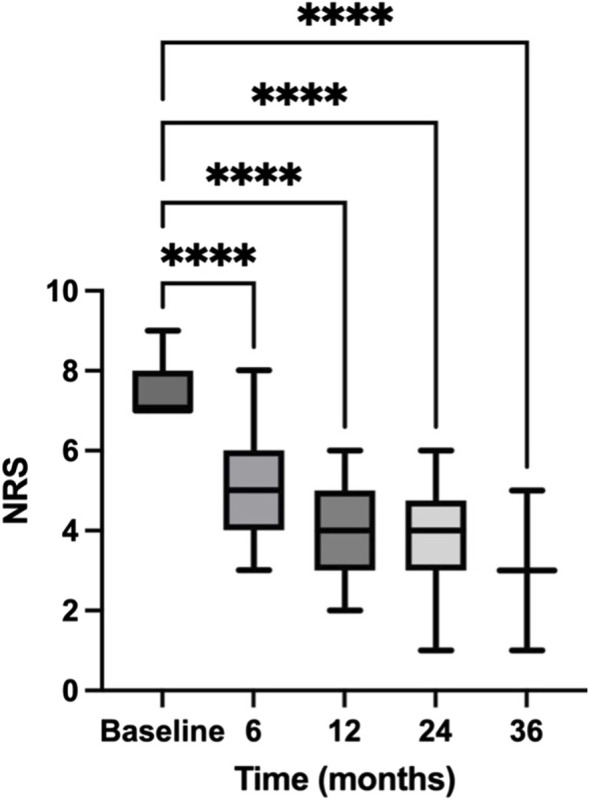
Temporal reduction in NRS scores for patients with moderate pain. This figure charts the trajectory of Numeric Rating Scale (NRS) scores for patients with moderate pain over a 36-month period. The NRS scores indicate patient-reported pain levels at baseline and subsequent intervals of 6, 12, 24, and 36 months, documenting the progression of pain relief in patients with moderate pain over the study period. Data are presented as mean ± standard error (SE). **** = *p* < 0.0001.

Patients with moderate pain are defined those with a NRS score ranging from 5 to 6. Data are presented in Figure 2. Initially, patients exhibited a significant reduction in pain from baseline (mean NRS = 5.76) to 6 months (mean NRS = 3.932), with a mean difference of 1.828 (95% CI: 1.521–2.135). This change was statistically significant (*p* < 0.00001), as indicated by Šídák’s multiple comparisons test, highlighting the immediate impact of the treatment regimen.

From 6 to 12 months, the pain scores further decreased, moving from a mean of 3.932 to 3.197, and achieving a mean difference of 0.7348 (95% CI: 0.4771–0.9926). This reduction was also significant (*p* < 0.00001), demonstrating continued effectiveness of the intervention over the medium term. The period from 12 to 24 months showed a slight, non-significant increase in pain scores (mean difference = −0.06494, 95% CI: −0.2663 to 0.1365), with a *p*-value of 0.8746. This stabilization suggests a plateau in the treatment effect or adaptation of the pain perception among patients. Between 24 and 36 months, the analysis indicated a non-significant change in pain levels with a mean difference of 1.190 (95% CI: −1.103–3.484) and a *p*-value of 0.1682. Despite this, the general trend from initial to final assessment shows a substantial overall decrease in pain levels.

Comparing NRS score between baseline and 36 months, we found a substantial reduction in pain levels over the 36-month period (*p* < 0.00001). The statistical analysis confirms that the decrease in pain scores is not only statistically significant but also clinically relevant, indicating the potential benefits of continued and consistent pain management strategies over extended periods.

### 3.3 NRS score in patients with severe pain

**FIGURE 3:**
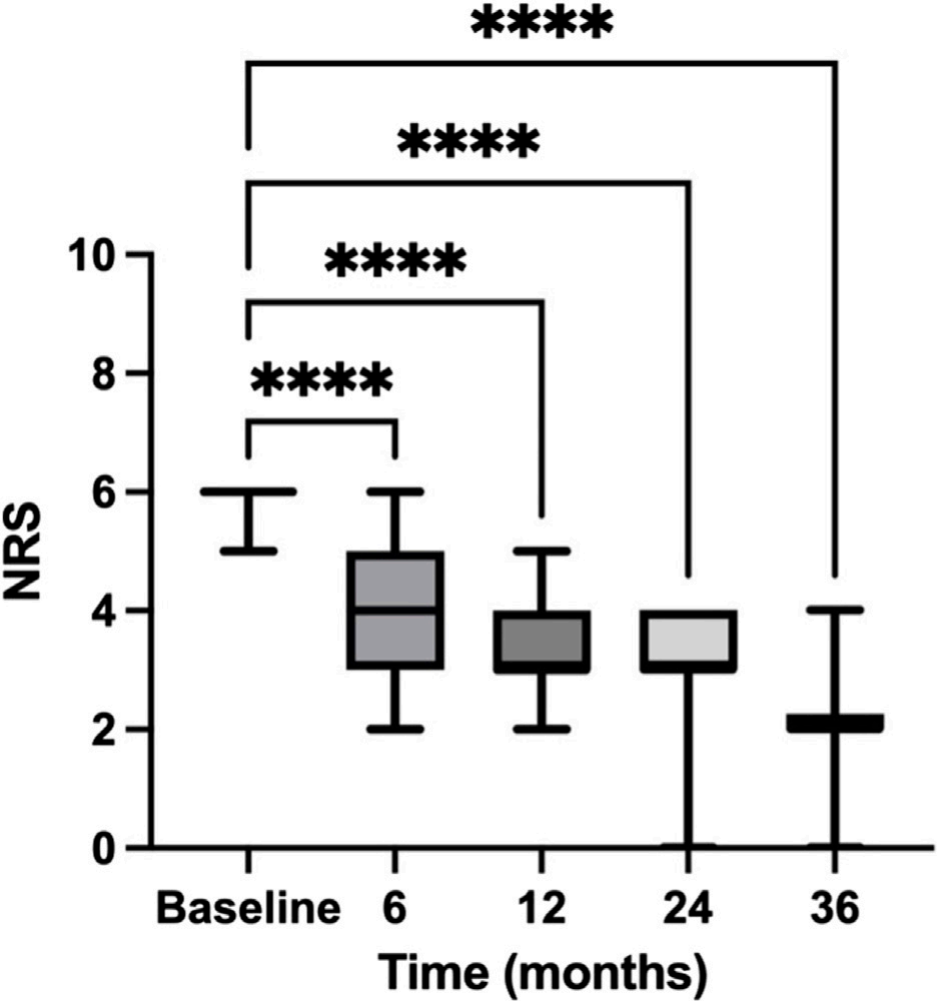
Temporal reduction in NRS scores for patients with severe pain. This figure charts the trajectory of Numeric Rating Scale (NRS) scores for patients with severe pain over a 36-month period. The NRS scores indicate patient-reported pain levels at baseline and subsequent intervals of 6, 12, 24, and 36 months, documenting the progression of pain relief in patients with severe pain over the study period. Data are presented as mean ± standard error (SE). **** = *p* < 0.0001.

Patients with severe pain are those with a NRS score equal to or greater than 6. Data are presented in Figure 3. Initially, patients reported a high average NRS score of 7.503 (±1.507 SE) at baseline, reflecting severe pain. By the 6-month mark, this score significantly decreased to 4.810, with a mean difference of 2.693 (95% CI: 2.445–2.940), and a *p*-value <0.00001. This substantial decrease indicates a strong initial response to the treatment. Further decreases were observed at subsequent intervals. From 6 to 12 months, the NRS score reduced to 4.132, showing a mean difference of 0.6784 (95% CI: 0.5309 to 0.8259, *p* < 0.00001). Between 12 and 24 months, a smaller yet significant reduction occurred, lowering the NRS to 3.902 (mean difference = 0.2302, 95% CI: 0.09596 to 0.3644, *p* = 0.00001). The most notable continued improvement was between 24 and 36 months. The NRS score further declined to 2.956, with a mean difference of 0.9459 (95% CI: 0.6724 to 1.219, *p* < 0.00001). This persistent decrease underscores the treatment’s sustained effectiveness over the long term.

By comparing NRS scores at baseline and 36 months, we observed a significant decline in pain intensity over the 36-month timeframe (*p* < 0.00001). As with patients with moderate pain, this reduction in pain scores is not just statistically noteworthy but also clinically meaningful, underscoring the potential advantages of maintaining consistent pain management strategies over prolonged durations.

### 3.4 Patients’ satisfaction (PGIC test)

**FIGURE 4:**
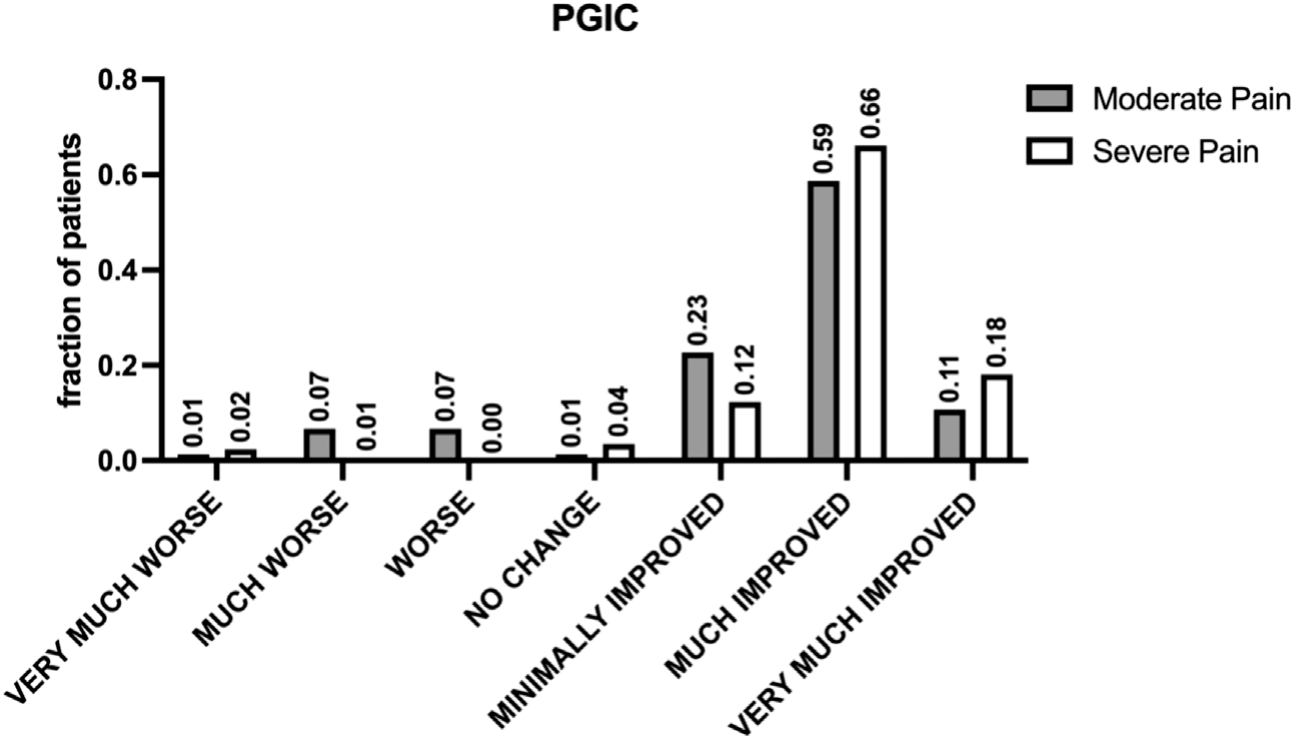
Patient-reported outcomes assessed by PGIC. The figure shows the results of the Patients’ Global Impression of Change (PGIC) scale, specifically designed to assess patients’ perceptions of change following treatment. This seven-point verbal scale offers options ranging from “very much improved” to “very much worsened,” including “much improved,” “minimally improved,” “no change,” “minimally worsened,” and “much worsened.” indicating high levels of patient satisfaction and perceived improvement in pain management across different pain intensities.

The PGIC results from this clinical retrospective study (Figure 4) reflect patient-reported outcomes, providing insights into their satisfaction with the treatment. In the study, patients with moderate pain showed significant improvements, with many reporting being “much improved” or “very much improved.” Specifically, out of the total respondents, 44 reported being “much improved” and 8 “very much improved.” Conversely, patients with severe pain, while also showing a significant number of positive responses, had a more varied spectrum of improvement. A substantial 113 patients reported feeling “much improved,” and 31 felt “very much improved.”

A contingency analysis of the PGIC data, evaluated through a chi-square test, confirmed the statistical significance of these observations with a *p*-value of 0.00002, indicating a strong association between the degree of pain relief and the initial severity of pain. This result underscores the effectiveness of buprenorphine in significantly improving patient outcomes across different pain intensities.

Additionally, a Mann-Whitney test comparing the distribution of PGIC scores between moderate and severe pain groups did not show a statistically significant difference (*p*-value = 0.6871), suggesting that buprenorphine’s effectiveness in improving patient satisfaction is consistent across these groups despite the severity of pain. Overall, the data from the PGIC scale indicates high levels of patient satisfaction with buprenorphine treatment for pain.

### 3.5 Longitudinal responder rates in pain management over 36 months

The secondary endpoint of our study was the evaluation of responder rates at 6, 12, 24, and 36 months, where responders were classified based on significant reductions in pain intensity as quantified by the NRS score (Figures 2, 3) and positive evaluations on the of PGIC (Figure 4). The data provided detailed changes in NRS scores and corresponding PGIC outcomes for patients with moderate and severe pain, which enabled a comprehensive assessment of treatment efficacy over the specified periods.

For patients with moderate pain, substantial improvements were consistently observed across all intervals. At 6 months, the mean NRS score significantly reduced from baseline, which continued to decrease through the 12-, and 24-month assessments. The largest reduction was noted from baseline to 6 months, with continuous albeit smaller improvements thereafter. At 36 months, despite the non-significant *p*-value, the overall trend indicated a marked improvement from the initial state. According to the PGIC, a majority of these patients reported improvements ranging from “minimally improved” to “very much improved,” with the peak improvement noted at 6 and 12 months.

In severe pain patients, the responder rate was even more pronounced. Starting with a higher baseline NRS score, the decrease by the 6-month mark was significant, continuing through to 36 months. The NRS scores indicated a steady and significant decrease in pain levels, with the most notable improvement between baseline and the final assessment. PGIC results mirrored these findings, with a significant number of patients reporting “much improved” to “very much improved” statuses, particularly notable in the later stages of the study period.

Overall, the percentage of responders, classified by significant NRS reductions and positive PGIC scores, consistently increased over time in both moderate and severe pain groups. The chi-square test confirmed the statistical significance of these observations across different intervals, illustrating the effectiveness of the treatment in managing pain and improving patient perceptions over the long term. This sustained improvement over 36 months highlights the potential of consistent pain management strategies to enhance patient outcomes significantly.

### 3.6 Buprenorphine’s efficacy in preventing opioid tolerance among moderate pain patients

To determine if tolerance developed in our study, we calculated the morphine-equivalent dose for each patient (as specified in the *Materials and Methods* section) at various time points—baseline, 6, 12, 24, and 36 months.

The dosages administered were as follows: at baseline, 5 mcg; at 6 months, 5.11 mcg; at 12 months, 9.32 mcg; at 24 months, 9.29 mcg; and at 36 months, 11.07 mcg. These dosages, when converted to morphine equivalents, resulted in: 12 mg at baseline, 12.246 mg at 6 months, 22.368 mg at 12 months, 22.296 mg at 24 months, and 26.568 mg at 36 months.

None of the calculated morphine-equivalent doses approached the FDA threshold of 60 mg/day (or the 25mcg/h of transdermal buprenorphine threshold), suggesting that none of the patients reached the opioid tolerance level under buprenorphine treatment throughout the 36-month study period.

**FIGURE 5:**
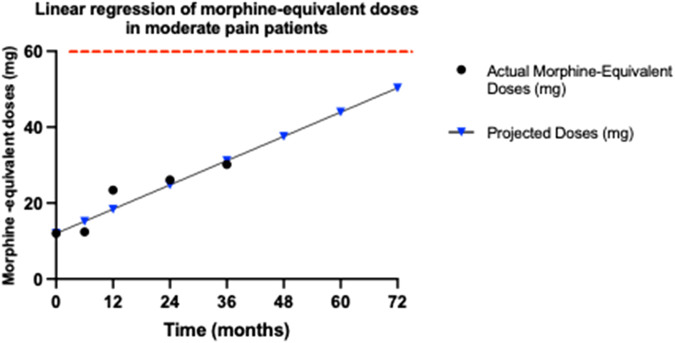
Trends in morphine-equivalent doses for moderate pain patients. This graph illustrates the morphine-equivalent doses, calculated from buprenorphine dosages over 36 months for patients with moderate pain, using an equianalgesic conversion factor, where 5 μg per hour of transdermal buprenorphine equates to 12 mg of morphine per day. The doses at each time point (baseline, 6, 12, 24, and 36 months) are plotted to assess any increase that might indicate opioid tolerance. The graph displays both the actual recorded doses until 36 months (black dots) and the projected morphine-equivalent doses (blue triangles) up to 72 months, in patients with moderate pain. The red dashed line indicates the opioid tolerance threshold of 60 mg/day, as defined by FDA. Linear regression analysis was employed to determine the slope of the dose trend, represented as mg/month, which quantifies the rate of increase in dosage requirements. The slope of 0.4117 mg/month suggests a very gradual increase in required dosage, remaining significantly below the 60 mg/day threshold associated with opioid tolerance, even at a projected period of 72 months.

Statistical analysis, particularly linear regression, was employed to trace the trajectory of doses over time (Figure 5). The findings indicated a gradual increase in the morphine-equivalent doses, reflected by a slope of 0.4117 mg/month. However, even at 36 months, the projected average dose remained substantially below the tolerance threshold, suggesting that the moderate pain patients did not develop significant opioid tolerance within the study period. This is particularly highlighted by the minimal increase observed between 12 and 36 months. To further analyze the trend of developing tolerance in patients with moderate pain, we projected the increase in buprenorphine dosage over 72 months. Even with this extended projection, the anticipated doses at 72 months remain below the 60 mg/day of morphine (or the 25mcg/h transdermal buprenorphine) tolerance threshold.

This indicates that buprenorphine is effective in managing moderate pain without leading to the development of opioid tolerance. This outcome highlights buprenorphine’s potential to provide sustained pain relief in patients with moderate pain, while minimizing the risk of tolerance, a significant advantage over other opioids where tolerance development is more common.

### 3.7 Buprenorphine’s efficacy in preventing opioid tolerance among severe pain patients

We also assessed the potential development of opioid tolerance in the long-term treatment of severe pain with buprenorphine. We performed the same analyses and calculations as in patients with moderate pain.

For severe pain patients, the dosages administered were as follows: at baseline, 5 mcg; at 6 months, 5.17 mcg; at 12 months, 9.71 mcg; at 24 months, 10.89 mcg; and at 36 months, 12.57 mcg. These dosages, when converted to morphine equivalents, resulted in: 12 mg at baseline, 12.408 mg at 6 months, 23.304 mg at 12 months, 26.136 mg at 24 months, and 30.168 mg at 36 months. Each of these morphine-equivalent doses remains well below the 60 mg threshold defined by the FDA for opioid tolerance. These findings indicate that none of the severe pain patients treated with buprenorphine developed opioid tolerance over the 36-month period.

**FIGURE 6:**
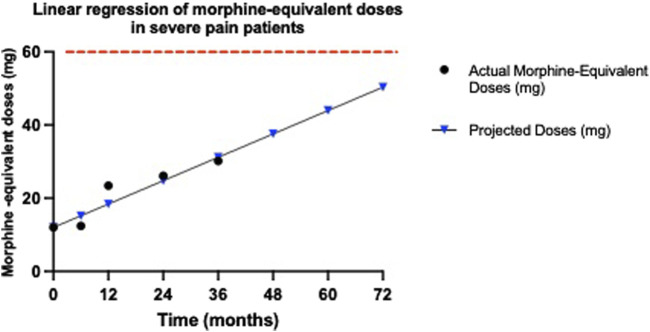
Morphine-equivalent dose trends for severe pain patients. This graph illustrates the morphine-equivalent doses derived from buprenorphine dosages for patients with severe pain, calculated using an equianalgesic conversion, where 5 μg per hour of transdermal buprenorphine corresponds to 12 mg of morphine per day. Dose trends are presented from baseline through 36 months to monitor for signs of opioid tolerance. The graph displays both the actual recorded doses until 36 months (black dots) and the projected morphine-equivalent doses (blue triangles) up to 72 months, in patients with moderate pain. The red dashed line indicates the opioid tolerance threshold of 60 mg/day, as defined by FDA. Linear regression analysis provides the slope of these trends, measured in mg/month, to evaluate the rate of increase in dosage requirements. The obtained slope of 0.5325 mg/month indicates a modest upward trend in required dosages. Despite this increase, the doses remain well below the FDA’s 60 mg/day threshold for opioid tolerance.

Moreover, despite a consistent uptrend in doses, depicted by a regression slope of 0.5325 mg/month, the results revealed that even after 36 months, the morphine-equivalent doses remained significantly below the critical threshold of 60 mg/day of morphine (or the 25 mcg/h of transdermal buprenorphine) (Figure 6). Even among patients with severe pain, the dosage increase observed between 12 and 36 months was minimal, further underscoring underscores the sustained efficacy of buprenorphine in providing long-term pain relief without significant tolerance development. Specifically, the projected dose at 36 months approached only 31.6629 mg, well under the threshold required for the development of opioid tolerance. To further investigate the trend of developing tolerance, we projected the increase in buprenorphine dosage over a 72-month period. Even with this projection, the anticipated doses at 72 months remain below the 60 mg/day tolerance threshold.

These results underscores buprenorphine’s efficacy not only in effectively managing severe pain but also in maintaining its effectiveness without escalating to tolerance. Such data affirm the suitability of buprenorphine for long-term management of severe pain, highlighting its potential as a key therapeutic in opioid stewardship initiatives aimed at reducing the risk of tolerance and dependence.

### 3.8 Safety

The following adverse events were reported (Table 2): constipation, reported by 1 patient (0.41% of the total cohort); skin reactions, reported by 6 patients, (2.44%); pruritus, experienced by 3 patients (1.22%); headache, reported by 3 patients (1.22%); nausea, reported by 2 patients (0.81%). No patients in this study reported issues as opioid use disorder or opioid abuse. These findings suggest that despite buprenorphine transdermal patches are generally well-tolerated, some patients may experience mild to moderate side effects, the most common of which are skin reactions.

**TABLE 2:** Adverse events reported in the study cohort. The table presents the adverse events reported during the study, including constipation, skin reactions, pruritus, headache, and nausea, along with the number of patients affected and the percentage relative to the total cohort.

Adverse event	Number of patients	Percentage of total cohort (%)
Constipation	1	0.41
Skin reactions	6	2.44
Pruritus	3	1.22
Headache	3	1.22
Nausea	2	0.81

## 4 Discussion

The findings from this retrospective study emphasize the sustained efficacy and reduced side effects of buprenorphine in managing moderate and severe chronic pain, reinforcing its role as a pivotal tool in the pain management spectrum. Notably, the study revealed a significant reduction in pain scores over a 36-month period, demonstrating buprenorphine’s potential for long-term use, without the significant risk of side effects and with a reduced tolerance development that is often associated with other opioids.

This is consistent with the body of research that highlights buprenorphine’s distinct advantages in pain management. For example, a multicenter, randomized, double-blind, placebo-controlled trial demonstrated that buprenorphine provides effective pain relief with stable dosing over time in patients with chronic non-cancer pain (Sittl et al., 2003). This stability in dosing is particularly important in the context of chronic pain management, as it reduces the risks associated with dose escalation, such as increased side effects and the potential for tolerance development. Davis and colleagues further highlight buprenorphine’s clinical benefits, noting its stable pharmacokinetic profile which is less influenced by patient-specific factors such as renal function (Davis et al., 2018). This aspect is particularly advantageous in elderly chronic pain populations, where such comorbidities are common. Additionally, buprenorphine’s unique receptor binding properties may be able to provide an analgesic effect without the high risk of abuse and tolerance seen with other opioids (Davis et al., 2018) Similarly, Infantino et al. elaborate on buprenorphine’s distinct pharmacological actions, such as its biased agonism at mu-opioid receptors and high affinity for opioid-like receptor 1, which contribute to its effectiveness in neuropathic pain, a condition often resistant to traditional opioids (Infantino et al., 2021). This is in line with the low incidence of side effects, such as constipation and skin reactions, noted in our study, supporting buprenorphine’s favorable safety profile, which is crucial for enhancing patient compliance and quality of life. The concept of biased or even protean agonism at opioid receptors may explain how certain receptor ligands might preferentially activate beneficial signaling pathways while avoiding those leading to adverse effects (Neto et al., 2020; Infantino et al., 2021). Particularly, the low incidence of gastrointestinal symptoms such as constipation and nausea is noteworthy, as these are often more prevalent with traditional opioid therapies. A possible explanation comes from a study by Neto et al. (2020), which detailed the genetic and pharmacological characteristics of opioid receptor interactions, According to these authors, the biased agonism of buprenorphine preferentially activates G-protein pathways over beta-arrestin pathways. This selective activation is crucial because beta-arrestin pathway activation is often linked to adverse gastrointestinal effects, including opioid-induced constipation. By minimizing beta-arrestin pathway activation, buprenorphine significantly reduces the likelihood of these side effects, such as nausea and costipation. Moreover, buprenorphine’s antagonistic action at KOR may also play a role in mitigating gastrointestinal side effects. KOR activation has been associated with dysphoria and diuresis, but its antagonism by buprenorphine could contribute to a more favorable side effect profile. Moreover, in this study, no patients reported issues as opioid use disorder and abuse, further highlighting buprenorphine’s favorable profile. Overall, our data indicate that the safety profile of buprenorphine transdermal patches appears favorable, with the majority of adverse events being minor and manageable.

Further examination of patient satisfaction, as measured by the PGIC, shows that most patients reported significant improvements in their pain and overall wellbeing. This aligns with previous findings, which highlight the patient-centered benefits of buprenorphine, particularly its ability to provide stable pain control with minimal side effects (Pergolizzi et al., 2010; Ehrlich and Darcq, 2018; Webster et al., 2020).

A pivotal finding from our study indicates that buprenorphine does not lead to significant tolerance development, both in patients with moderate and severe pain. According to FDA guidelines (as defined in the fentanyl official labeling), opioid tolerance is defined as needing 60 mg/day of morphine or an equivalent dose of another opioid for a week or more. (Dowell et al., 2022; “Rx only -Fentanyl Full Prescribing Information”) In our study, even after 36 months, the morphine-equivalent doses of buprenorphine remained well below this threshold, with only a minor increase in dosage observed between 12 and 36 months. This stability in dosing highlights the sustained efficacy of buprenorphine, minimizing the risk of tolerance development. This outcome contrasts sharply with typical results seen in patients treated with other long-term opioids, where tolerance development often necessitates progressively larger doses to achieve the same pain relief effect (Volkow and Blanco, 2021). Our findings suggest that buprenorphine maintains its efficacy over long periods without the need for significant dose escalations. The gentle upward trajectory of the dosage increase, even at 36 months, suggests that the increases were minimal and remained well below the threshold that would indicate significant tolerance. This slow rate of increase, especially between 12 and 36 months, further underscores the drug’s resilience against the typical rapid tolerance development seen with other opioids. Furthermore, when extrapolated to a 72-month period, the dosages delivered via buprenorphine transdermal patches remain significantly below the 60 mg/day morphine-equivalent threshold, both in patients with moderate and severe pain, which would suggest the development of opioid tolerance.

From our data it appears that the choice of transdermal patches for opioid delivery offers a winning strategy in pain management, due to several key pharmacological and patient compliance factors. Transdermal buprenorphine provides a controlled, continuous delivery of the medication over a 7-day period, ensuring stable plasma drug concentrations and minimizing fluctuations that can lead to breakthrough pain or side effects associated with peak levels (Plosker, 2011). This consistent delivery helps in managing pain effectively without the peaks and troughs associated with oral or sublingual forms, which can enhance patient compliance and overall pain management. The pharmacokinetics of transdermal buprenorphine minimize drug-drug interactions and reduce the incidence of central nervous system side effects such as cognitive impairment and sedation, which are often seen with other opioids. This is especially beneficial in elderly patients or those with renal and hepatic impairments, where managing side effects and interactions becomes crucial (Owsiany et al., 2019; Zhuo et al., 2021). Additionally, there is a ceiling effect for respiratory depression, enhancing the safety profile of buprenorphine, especially in a population at risk of opioid overdosage (Plosker, 2011; Owsiany et al., 2019; Neto et al., 2020; Infantino et al., 2021).

Our retrospective study offers valuable real-world insights on buprenorphine’s long-term efficacy in managing chronic pain in both moderate and severe pain patients, but come with inherent limitations. Selection bias can occur since data are limited to existing medical records, potentially overlooking pertinent information. Issues like recall bias from inconsistent records, uncontrolled confounding variables, and variability in data quality can affect the study’s reliability. Additionally, the lack of randomization means our data can suggest associations but not establish causality. Despite these limitations, our study benefits from a large sample size and longitudinal data spanning 3 years, which enhances the reliability of the findings and their applicability in clinical settings. The specificity of data on morphine-equivalent doses and the comprehensive measures of both efficacy and safety provide a comprehensive view of buprenorphine’s role in pain management. These strengths underscore the usefulness of buprenorphine in clinical practice while highlighting the necessity of cautious interpretation of results due to the study’s retrospective nature. For these reasons, while our retrospective design inherently limits the ability to draw causal inferences, the use of advanced statistical controls and long-term follow-up contribute to a robust dataset from which meaningful conclusions can be drawn about buprenorphine’s efficacy and safety.

In conclusion, our findings support recent guidelines by health authorities advocating for the expanded use of buprenorphine in chronic pain management. This study provides empirical support for such policies, emphasizing buprenorphine’s lower risk profile compared to traditional opioids. These findings advocate for broader clinical use and potential policy shifts to favor buprenorphine as a frontline treatment in chronic pain management strategies, thereby improving patient outcomes and addressing critical aspects of the worldwide opioid crisis.

## Data Availability

The original contributions presented in the study are included in the article/supplementary material, further inquiries can be directed to the corresponding author.
