# Corrigendum: Long-term efficacy and reduced side-effects of buprenorphine in patients with moderate and severe chronic pain

**DOI:** 10.3389/fphar.2024.1491886

**Published:** 2024-09-30

**Authors:** Alfonso Papa, Anna Maria Salzano, Maria Teresa Di Dato, Vincenzo Desiderio, Pietro Buonavolontà, Pietro Mango, Elisabetta Saracco, Dario Tammaro, Livio Luongo, Sabatino Maione

**Affiliations:** ^1^ Department of Pain Management—AO “Ospedale dei Colli”–Monaldi Hospital, Napoli, Italy; ^2^ Department of Experimental Medicine, University of Campania “Luigi Vanvitelli”, Naples, Italy; ^3^ Department of Experimental Medicine, Division of Pharmacology, University of Campania “Luigi Vanvitelli”, Naples, Italy

**Keywords:** chronic pain, opioids, tolerance, ransdermal patches, pain relief, opioid crisis

In the published article, there was an error in Figures 1, 4, 5 as published. The figures were mismatched with their respective images.

Figure 1 should display the image originally intended for Figure 5; the caption remains correct. The corrected Figure 1 and its caption appear below.

**FIGURE 1:**
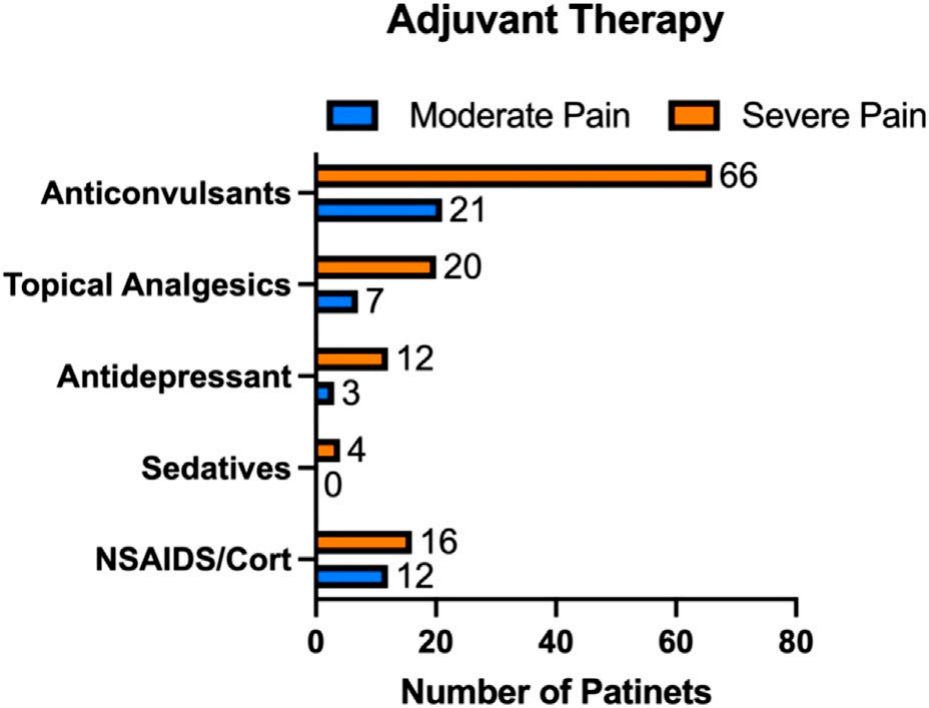
Additional therapies prescribed alongside buprenorphine for chronic pain management. Among the 248 patients enrolled, 118 with severe pain and 34 with moderate pain were prescribed adjuvant treatments, including anticonvulsants, topical agents, antidepressants, sedatives, and NSAIDs/corticosteroids.

Figure 4 should display the image originally intended for Figure 1; the caption remains correct. The corrected Figure 4 and its caption appear below.

**FIGURE 4:**
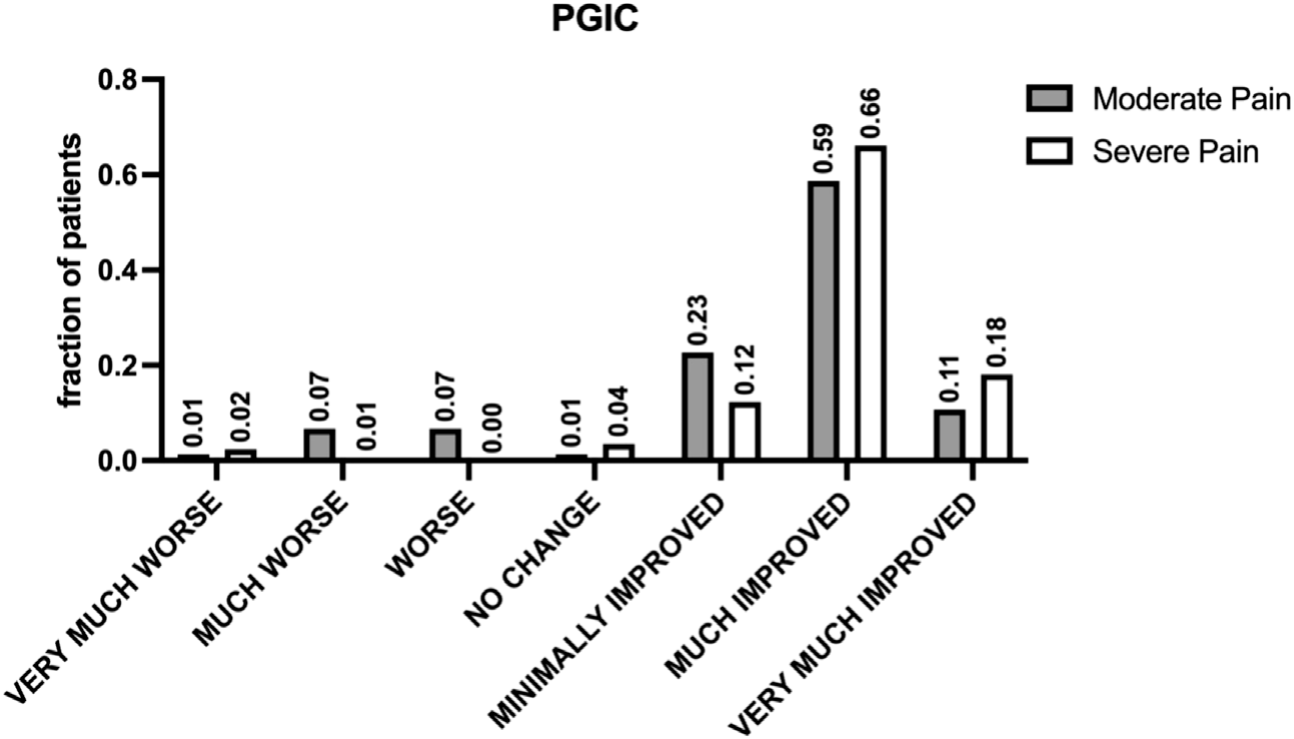
Patient-reported outcomes assessed by PGIC. The figure shows the results of the Patients’ Global Impression of Change (PGIC) scale, specifically designed to assess patients’ perceptions of change following treatment. This seven-point verbal scale offers options ranging from “very much improved” to “very much worsened,” including “much improved,” “minimally improved,” “no change,” “minimally worsened,” and “much worsened.” indicating high levels of patient satisfaction and perceived improvement in pain management across different pain intensities.

Figure 5 should display the image originally intended for Figure 4; the caption remains correct. The corrected Figure 5 and its caption appear below.

**FIGURE 5:**
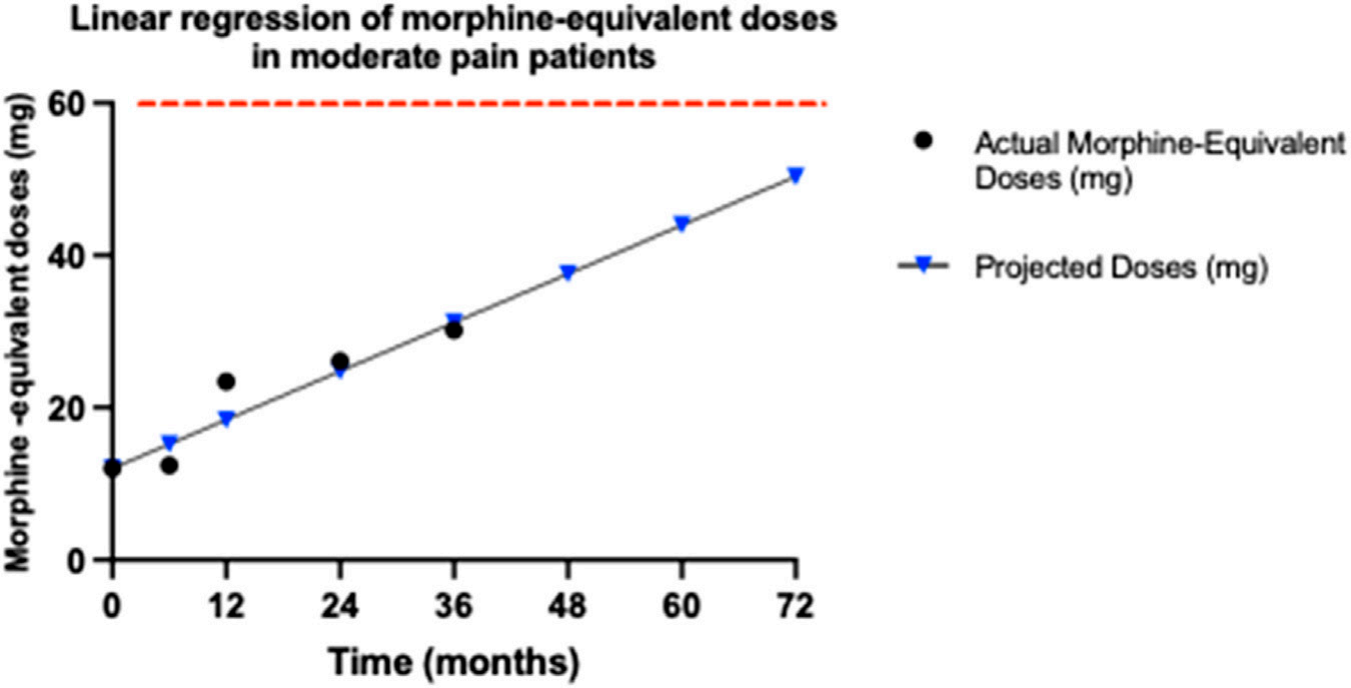
Trends in morphine-equivalent doses for moderate pain patients. This graph illustrates the morphine-equivalent doses, calculated from buprenorphine dosages over 36 months for patients with moderate pain, using an equianalgesic conversion factor, where 5 μg per hour of transdermal buprenorphine equates to 12 mg of morphine per day. The doses at each time point (baseline, 6, 12, 24, and 36 months) are plotted to assess any increase that might indicate opioid tolerance. The graph displays both the actual recorded doses until 36 months (black dots) and the projected morphine-equivalent doses (blue triangles) up to 72 months, in patients with moderate pain. The red dashed line indicates the opioid tolerance threshold of 60 mg/day, as defined by FDA. Linear regression analysis was employed to determine the slope of the dose trend, represented as mg/month, which quantifies the rate of increase in dosage requirements. The slope of 0.4117 mg/month suggests a very gradual increase in required dosage, remaining significantly below the 60 mg/day threshold associated with opioid tolerance, even at a projected period of 72 months.

The authors apologize for this error and state that this does not change the scientific conclusions of the article in any way. The original article has been updated.

